# Supplementary material for: Single Cell Transcriptomic and Chromatin Profiles Suggest Layer Vb Is the Only Layer With Shared Excitatory Cell Types in the Medial and Lateral Entorhinal Cortex
Source: Front Neural Circuits. 2022 Jan 26;15:806154. doi: 10.3389/fncir.2021.806154 (PMC8826650; doi:10.3389/fncir.2021.806154)
Supplement: Supplementary file 14 [file Data_Sheet_1.ZIP › 16/knownResults.html]

16 - Homer Known Motif Enrichment Results


# Homer Known Motif Enrichment Results (16)

Homer *de novo* Motif Results  
Gene Ontology Enrichment Results  
Known Motif Enrichment Results (txt file)  
Total Target Sequences = 1000, Total Background Sequences = 48048

|  |  |  |  |  |  |  |  |  |  |  |  |
| --- | --- | --- | --- | --- | --- | --- | --- | --- | --- | --- | --- |
| Rank | Motif | Name | P-value | log P-pvalue | q-value (Benjamini) | # Target Sequences with Motif | % of Targets Sequences with Motif | # Background Sequences with Motif | % of Background Sequences with Motif | Motif File | SVG |
| 1 | A G C T A C G T A C T G A T G C A G T C C G T A C T G A T A C G | NF1-halfsite(CTF)/LNCaP-NF1-ChIP-Seq(Unpublished)/Homer | 1e-39 | -9.125e+01 | 0.0000 | 403.0 | 40.30% | 10402.3 | 21.65% | motif file (matrix) | svg |
| 2 | C T G A C T A G A C T G G C A T C T A G G C A T A T C G C T G A C G T A G T C A | Tbx21(T-box)/GM12878-TBX21-ChIP-Seq(Encode)/Homer | 1e-29 | -6.712e+01 | 0.0000 | 278.0 | 27.80% | 6740.6 | 14.03% | motif file (matrix) | svg |
| 3 | C T G A C T A G A T C G G C A T A C T G G T A C A T G C C G T A A C T G G C T A A G T C C G T A | Tbox:Smad(T-box,MAD)/ESCd5-Smad2\_3-ChIP-Seq(GSE29422)/Homer | 1e-27 | -6.249e+01 | 0.0000 | 94.0 | 9.40% | 1174.4 | 2.44% | motif file (matrix) | svg |
| 4 | C T G A C T G A C A T G A T C G A G C T A T C G G A C T C A T G C T G A G T C A | Tbr1(T-box)/Cortex-Tbr1-ChIP-Seq(GSE71384)/Homer | 1e-25 | -5.855e+01 | 0.0000 | 347.0 | 34.70% | 9772.9 | 20.34% | motif file (matrix) | svg |
| 5 | C T A G C T G A T C A G A T C G A G C T C A T G G A C T A G T C C T G A T G C A | Tbx6(T-box)/ESC-Tbx6-ChIP-Seq(GSE93524)/Homer | 1e-25 | -5.798e+01 | 0.0000 | 261.0 | 26.10% | 6518.3 | 13.57% | motif file (matrix) | svg |
| 6 | C A T G G T A C G A C T G C T A C G T A C G T A C G T A G C T A G A C T C T G A T C A G G T A C | Mef2c(MADS)/GM12878-Mef2c-ChIP-Seq(GSE32465)/Homer | 1e-23 | -5.434e+01 | 0.0000 | 172.0 | 17.20% | 3590.1 | 7.47% | motif file (matrix) | svg |
| 7 | C A T G A G T C A G C T C G T A C G A T C G A T G C A T G C A T C G A T C T G A C A T G T G A C | Mef2d(MADS)/Retina-Mef2d-ChIP-Seq(GSE61391)/Homer | 1e-22 | -5.282e+01 | 0.0000 | 94.0 | 9.40% | 1348.3 | 2.81% | motif file (matrix) | svg |
| 8 | C T G A C T A G A T C G A G C T A C T G G A C T A G T C C T G A | Tbx5(T-box)/HL1-Tbx5.biotin-ChIP-Seq(GSE21529)/Homer | 1e-22 | -5.276e+01 | 0.0000 | 576.0 | 57.60% | 20124.6 | 41.88% | motif file (matrix) | svg |
| 9 | G C T A G A C T G A C T T G C A C G T A A G T C C G T A T A G C G A T C G A C T | Eomes(T-box)/H9-Eomes-ChIP-Seq(GSE26097)/Homer | 1e-19 | -4.441e+01 | 0.0000 | 461.0 | 46.10% | 15488.8 | 32.24% | motif file (matrix) | svg |
| 10 | C A T G A G T C G A C T C G T A C G A T G C A T G A C T G C A T C G A T C T A G C A T G T G A C | Mef2b(MADS)/HEK293-Mef2b.V5-ChIP-Seq(GSE67450)/Homer | 1e-18 | -4.302e+01 | 0.0000 | 245.0 | 24.50% | 6652.1 | 13.84% | motif file (matrix) | svg |
| 11 | G T A C G A C T C G T A C T G A T C G A C G T A G C T A C A G T C T G A T A C G | Mef2a(MADS)/HL1-Mef2a.biotin-ChIP-Seq(GSE21529)/Homer | 1e-17 | -3.948e+01 | 0.0000 | 141.0 | 14.10% | 3111.0 | 6.47% | motif file (matrix) | svg |
| 12 | G A C T C T A G A T G C A G T C G T C A T A C G A T G C A T C G | HIC1(Zf)/Treg-ZBTB29-ChIP-Seq(GSE99889)/Homer | 1e-15 | -3.508e+01 | 0.0000 | 361.0 | 36.10% | 11852.2 | 24.67% | motif file (matrix) | svg |
| 13 | C G T A C T A G C A T G A G C T A C T G C G A T A T C G C G T A G T C A G T C A | Tbet(T-box)/CD8-Tbet-ChIP-Seq(GSE33802)/Homer | 1e-15 | -3.486e+01 | 0.0000 | 251.0 | 25.10% | 7355.8 | 15.31% | motif file (matrix) | svg |
| 14 | C T A G T C G A A C G T A C T G C G T A A T G C A C G T G T A C C G T A A G C T G A T C G T A C | Atf3(bZIP)/GBM-ATF3-ChIP-Seq(GSE33912)/Homer | 1e-13 | -2.997e+01 | 0.0000 | 158.0 | 15.80% | 4118.1 | 8.57% | motif file (matrix) | svg |
| 15 | A C T G C T A G T C G A C G A T C A T G G C T A A T C G C G A T G T A C G C T A A G C T G T A C | Fra1(bZIP)/BT549-Fra1-ChIP-Seq(GSE46166)/Homer | 1e-11 | -2.569e+01 | 0.0000 | 133.0 | 13.30% | 3438.2 | 7.16% | motif file (matrix) | svg |
| 16 | A G T C G A C T C A G T A C T G C T A G T G A C G C T A A T G C G C A T A T C G C G A T A C T G G A T C G T A C G T C A C T G A | NF1(CTF)/LNCAP-NF1-ChIP-Seq(Unpublished)/Homer | 1e-10 | -2.483e+01 | 0.0000 | 85.0 | 8.50% | 1838.2 | 3.83% | motif file (matrix) | svg |
| 17 | C T A G T C G A G C A T C A T G G C T A T A G C C G A T G T A C C T G A A G C T | JunB(bZIP)/DendriticCells-Junb-ChIP-Seq(GSE36099)/Homer | 1e-10 | -2.467e+01 | 0.0000 | 130.0 | 13.00% | 3383.1 | 7.04% | motif file (matrix) | svg |
| 18 | C A T G C T A G T C G A A C G T A C T G C G T A T A G C C G A T T G A C C G T A A G C T G A T C | Fra2(bZIP)/Striatum-Fra2-ChIP-Seq(GSE43429)/Homer | 1e-10 | -2.455e+01 | 0.0000 | 113.0 | 11.30% | 2788.9 | 5.80% | motif file (matrix) | svg |
| 19 | T C G A A C G T C A T G G C T A T A G C C G A T G T A C G C T A A C G T A T G C | AP-1(bZIP)/ThioMac-PU.1-ChIP-Seq(GSE21512)/Homer | 1e-10 | -2.403e+01 | 0.0000 | 164.0 | 16.40% | 4676.5 | 9.73% | motif file (matrix) | svg |
| 20 | C T A G T C G A A C G T A C T G C G T A T A G C C G A T G T A C C G T A A G C T G A T C G T A C | Jun-AP1(bZIP)/K562-cJun-ChIP-Seq(GSE31477)/Homer | 1e-10 | -2.393e+01 | 0.0000 | 66.0 | 6.60% | 1277.8 | 2.66% | motif file (matrix) | svg |
| 21 | A C T G C A T G T C G A A C G T A C T G C G T A A T C G A C G T G T A C C G T A G A C T A G T C | Fos(bZIP)/TSC-Fos-ChIP-Seq(GSE110950)/Homer | 1e-10 | -2.339e+01 | 0.0000 | 135.0 | 13.50% | 3633.7 | 7.56% | motif file (matrix) | svg |
| 22 | C T A G A G C T G A C T C A T G A G T C A G T C G T C A C A G T C T A G T C A G G T A C C T G A T C G A G A T C T G A C | Rfx2(HTH)/LoVo-RFX2-ChIP-Seq(GSE49402)/Homer | 1e-9 | -2.254e+01 | 0.0000 | 33.0 | 3.30% | 415.4 | 0.86% | motif file (matrix) | svg |
| 23 | C A G T T G C A A C G T A C T G C G T A A T C G C G A T T G A C C G T A A C G T | BATF(bZIP)/Th17-BATF-ChIP-Seq(GSE39756)/Homer | 1e-9 | -2.244e+01 | 0.0000 | 149.0 | 14.90% | 4208.3 | 8.76% | motif file (matrix) | svg |
| 24 | A G T C A T C G C T A G A G C T G A C T C T A G A G T C A G T C G C T A C A G T T C A G T C A G G A T C C T G A T C G A G A T C | RFX(HTH)/K562-RFX3-ChIP-Seq(SRA012198)/Homer | 1e-9 | -2.110e+01 | 0.0000 | 29.0 | 2.90% | 347.9 | 0.72% | motif file (matrix) | svg |
| 25 | C T A G T C G A C G A T A C T G C G T A T A C G A G C T T G A C G C T A A C G T G A T C T A G C | Fosl2(bZIP)/3T3L1-Fosl2-ChIP-Seq(GSE56872)/Homer | 1e-7 | -1.750e+01 | 0.0000 | 75.0 | 7.50% | 1808.6 | 3.76% | motif file (matrix) | svg |
| 26 | A G C T G C A T A C T G A C G T A G T C A C G T C T A G T A C G | Smad3(MAD)/NPC-Smad3-ChIP-Seq(GSE36673)/Homer | 1e-6 | -1.578e+01 | 0.0000 | 491.0 | 49.10% | 19701.1 | 41.00% | motif file (matrix) | svg |
| 27 | A T G C G A C T A C T G C A G T G A T C A C G T T A C G T A C G | Smad2(MAD)/ES-SMAD2-ChIP-Seq(GSE29422)/Homer | 1e-5 | -1.306e+01 | 0.0000 | 276.0 | 27.60% | 10285.8 | 21.41% | motif file (matrix) | svg |
| 28 | C G T A C G A T G A C T G A C T T G A C C T G A A T G C C T G A T A G C A G T C A C G T T C G A C A T G T A C G G A C T A T C G G A C T A C G T C T G A T C G A C G T A | Brachyury(T-box)/Mesoendoderm-Brachyury-ChIP-exo(GSE54963)/Homer | 1e-5 | -1.295e+01 | 0.0000 | 70.0 | 7.00% | 1861.4 | 3.87% | motif file (matrix) | svg |
| 29 | A G T C C G A T A C T G A T C G T G A C G C T A C A T G A T C G T G A C C G A T A C T G T A G C G T A C G T C A | Tlx?(NR)/NPC-H3K4me1-ChIP-Seq(GSE16256)/Homer | 1e-5 | -1.291e+01 | 0.0000 | 83.0 | 8.30% | 2337.8 | 4.87% | motif file (matrix) | svg |
| 30 | C T G A T C A G G T A C G C T A A C T G T G A C G C A T C A T G | SCL(bHLH)/HPC7-Scl-ChIP-Seq(GSE13511)/Homer | 1e-5 | -1.186e+01 | 0.0001 | 647.0 | 64.70% | 27834.3 | 57.93% | motif file (matrix) | svg |
| 31 | C G A T T A C G T G A C G A C T C A T G C G T A T A C G A C G T G T A C C T G A | Bach2(bZIP)/OCILy7-Bach2-ChIP-Seq(GSE44420)/Homer | 1e-4 | -1.052e+01 | 0.0004 | 45.0 | 4.50% | 1109.9 | 2.31% | motif file (matrix) | svg |
| 32 | C A T G C T A G A G C T G A C T C A T G A G T C G A T C G C T A C G A T C T A G T C A G G T A C C T G A T C G A | X-box(HTH)/NPC-H3K4me1-ChIP-Seq(GSE16256)/Homer | 1e-4 | -1.040e+01 | 0.0004 | 30.0 | 3.00% | 622.7 | 1.30% | motif file (matrix) | svg |
| 33 | A T G C T C G A A G T C A G C T A C G T G T A C A G T C G C T A C T A G C A T G G T C A C T G A T C A G A G T C | Stat3+il21(Stat)/CD4-Stat3-ChIP-Seq(GSE19198)/Homer | 1e-4 | -1.022e+01 | 0.0005 | 142.0 | 14.20% | 4888.7 | 10.17% | motif file (matrix) | svg |
| 34 | T G A C A G T C C T G A T G A C C G T A A C G T A C G T A G T C A G T C C G T A | TEAD1(TEAD)/HepG2-TEAD1-ChIP-Seq(Encode)/Homer | 1e-4 | -9.454e+00 | 0.0010 | 195.0 | 19.50% | 7222.5 | 15.03% | motif file (matrix) | svg |
| 35 | G A C T C A G T A G C T C G A T A G T C G A T C A G T C C G T A A T G C T C A G | Rbpj1(?)/Panc1-Rbpj1-ChIP-Seq(GSE47459)/Homer | 1e-4 | -9.406e+00 | 0.0010 | 248.0 | 24.80% | 9548.0 | 19.87% | motif file (matrix) | svg |
| 36 | T A C G A T C G T A G C G A T C A C T G A C G T A G T C A C G T C T A G A T C G | Smad4(MAD)/ESC-SMAD4-ChIP-Seq(GSE29422)/Homer | 1e-4 | -9.323e+00 | 0.0011 | 266.0 | 26.60% | 10359.8 | 21.56% | motif file (matrix) | svg |
| 37 | T A G C T C A G C A T G G C A T A G C T C G A T A T G C C G T A C G T A G T C A | CHR(?)/Hela-CellCycle-Expression/Homer | 1e-3 | -9.098e+00 | 0.0013 | 144.0 | 14.40% | 5093.6 | 10.60% | motif file (matrix) | svg |
| 38 | T C A G A G C T A C G T A C G T G T A C G A T C C G T A C T A G C A T G G T C A C G T A T C G A | STAT4(Stat)/CD4-Stat4-ChIP-Seq(GSE22104)/Homer | 1e-3 | -8.887e+00 | 0.0016 | 191.0 | 19.10% | 7124.1 | 14.83% | motif file (matrix) | svg |
| 39 | A G C T A T G C G A C T G C A T C G T A A G C T G T A C C G A T A T C G A G T C | Gata6(Zf)/HUG1N-GATA6-ChIP-Seq(GSE51936)/Homer | 1e-3 | -8.620e+00 | 0.0020 | 195.0 | 19.50% | 7334.7 | 15.27% | motif file (matrix) | svg |
| 40 | A G C T A G T C A T G C A G C T A C G T C G T A A C G T A G T C C G A T A T G C | Gata2(Zf)/K562-GATA2-ChIP-Seq(GSE18829)/Homer | 1e-3 | -8.405e+00 | 0.0025 | 140.0 | 14.00% | 5005.8 | 10.42% | motif file (matrix) | svg |
| 41 | C G T A C G T A C G T A G C A T G C A T T A C G G T A C G A C T A C T G C G T A A T C G A C G T G T A C C G T A A G C T | Bach1(bZIP)/K562-Bach1-ChIP-Seq(GSE31477)/Homer | 1e-3 | -8.276e+00 | 0.0027 | 18.0 | 1.80% | 328.8 | 0.68% | motif file (matrix) | svg |
| 42 | A C T G T C A G A G C T G A C T C A T G A G T C A G T C G C T A C G A T C T A G T C A G G T A C C T G A T C G A | Rfx1(HTH)/NPC-H3K4me1-ChIP-Seq(GSE16256)/Homer | 1e-3 | -8.243e+00 | 0.0028 | 41.0 | 4.10% | 1085.3 | 2.26% | motif file (matrix) | svg |
| 43 | C A G T T C A G A G C T G A C T A C G T A G T C G A T C G A C T C T G A A C T G G A T C C G T A C T G A A G T C G T A C | Rfx6(HTH)/Min6b1-Rfx6.HA-ChIP-Seq(GSE62844)/Homer | 1e-3 | -8.113e+00 | 0.0031 | 180.0 | 18.00% | 6753.4 | 14.06% | motif file (matrix) | svg |
| 44 | T C G A C T G A T A G C T G A C T C A G T C A G C G T A C G T A T C A G A G C T | ETV1(ETS)/GIST48-ETV1-ChIP-Seq(GSE22441)/Homer | 1e-3 | -7.862e+00 | 0.0039 | 224.0 | 22.40% | 8719.1 | 18.15% | motif file (matrix) | svg |
| 45 | T C G A T A G C G T C A A C T G A C T G C G T A C G T A C T A G A G C T T C A G | ERG(ETS)/VCaP-ERG-ChIP-Seq(GSE14097)/Homer | 1e-3 | -7.459e+00 | 0.0056 | 268.0 | 26.80% | 10755.0 | 22.38% | motif file (matrix) | svg |
| 46 | A G T C C T G A A G T C C G A T C A G T G A T C A T G C A C T G A T C G G A C T | Fli1(ETS)/CD8-FLI-ChIP-Seq(GSE20898)/Homer | 1e-3 | -7.298e+00 | 0.0065 | 174.0 | 17.40% | 6608.4 | 13.75% | motif file (matrix) | svg |
| 47 | T C G A T C G A A G T C C G T A C T A G T A G C A C G T A C T G | MyoG(bHLH)/C2C12-MyoG-ChIP-Seq(GSE36024)/Homer | 1e-3 | -6.953e+00 | 0.0089 | 161.0 | 16.10% | 6093.0 | 12.68% | motif file (matrix) | svg |
| 48 | A G C T C T A G T G A C C G T A A C G T C G A T A G T C A G T C C T G A C A T G | TEAD3(TEA)/HepG2-TEAD3-ChIP-Seq(Encode)/Homer | 1e-2 | -6.815e+00 | 0.0101 | 217.0 | 21.70% | 8579.6 | 17.86% | motif file (matrix) | svg |
| 49 | A C G T T C G A T C G A A G T C G T C A T A C G A T G C A C G T A C T G A G C T | Myf5(bHLH)/GM-Myf5-ChIP-Seq(GSE24852)/Homer | 1e-2 | -6.655e+00 | 0.0116 | 115.0 | 11.50% | 4162.8 | 8.66% | motif file (matrix) | svg |
| 50 | T A C G A T G C G C T A A C T G C G T A A C G T C G T A C T G A T A C G T C G A | Gata4(Zf)/Heart-Gata4-ChIP-Seq(GSE35151)/Homer | 1e-2 | -6.653e+00 | 0.0116 | 201.0 | 20.10% | 7895.4 | 16.43% | motif file (matrix) | svg |
| 51 | C T A G T C G A C G A T C T A G G C A T C A G T C T A G G A T C C G T A G T C A | CEBP:AP1(bZIP)/ThioMac-CEBPb-ChIP-Seq(GSE21512)/Homer | 1e-2 | -6.383e+00 | 0.0146 | 140.0 | 14.00% | 5266.8 | 10.96% | motif file (matrix) | svg |
| 52 | T A G C G T A C C G T A C T A G A C T G T G C A C G T A A T G C C G T A A T C G | AR-halfsite(NR)/LNCaP-AR-ChIP-Seq(GSE27824)/Homer | 1e-2 | -6.249e+00 | 0.0164 | 586.0 | 58.60% | 25950.4 | 54.01% | motif file (matrix) | svg |
| 53 | T C G A T A G C T G C A A C T G A C T G C G T A C G T A C T A G G A C T T A C G | ETS1(ETS)/Jurkat-ETS1-ChIP-Seq(GSE17954)/Homer | 1e-2 | -6.247e+00 | 0.0164 | 175.0 | 17.50% | 6813.6 | 14.18% | motif file (matrix) | svg |
| 54 | T A G C G C T A A C T G C G T A A C G T C G T A C G T A T A C G T C A G T C G A | Gata1(Zf)/K562-GATA1-ChIP-Seq(GSE18829)/Homer | 1e-2 | -6.143e+00 | 0.0175 | 118.0 | 11.80% | 4355.1 | 9.06% | motif file (matrix) | svg |
| 55 | A T G C A G T C G T A C A G C T T C G A C T A G G A T C C T G A G T C A A G T C G C T A T C A G | Rfx5(HTH)/GM12878-Rfx5-ChIP-Seq(GSE31477)/Homer | 1e-2 | -5.997e+00 | 0.0199 | 76.0 | 7.60% | 2613.6 | 5.44% | motif file (matrix) | svg |
| 56 | A G C T C A T G G C A T G A T C T G C A C T A G G A T C A C G T | Tgif2(Homeobox)/mES-Tgif2-ChIP-Seq(GSE55404)/Homer | 1e-2 | -5.847e+00 | 0.0227 | 506.0 | 50.60% | 22194.1 | 46.19% | motif file (matrix) | svg |
| 57 | G A C T C G A T T C A G G A T C G A C T A G C T A G C T A G T C G A T C C G T A C T A G C T A G T C G A T C G A C T G A | Bcl6(Zf)/Liver-Bcl6-ChIP-Seq(GSE31578)/Homer | 1e-2 | -5.846e+00 | 0.0227 | 266.0 | 26.60% | 10969.2 | 22.83% | motif file (matrix) | svg |
| 58 | C T G A A C G T A C G T A C G T A G T C G A C T C G A T C T G A A C T G C G T A C G T A T C G A | STAT5(Stat)/mCD4+-Stat5-ChIP-Seq(GSE12346)/Homer | 1e-2 | -5.636e+00 | 0.0270 | 77.0 | 7.70% | 2693.8 | 5.61% | motif file (matrix) | svg |
| 59 | A G T C A C G T A C T G A G C T A C G T A C G T G T C A A G T C | Foxo1(Forkhead)/RAW-Foxo1-ChIP-Seq(Fan\_et\_al.)/Homer | 1e-2 | -5.605e+00 | 0.0274 | 327.0 | 32.70% | 13824.6 | 28.77% | motif file (matrix) | svg |
| 60 | C G T A C T G A C T A G C G T A C G T A A G T C C G T A C A G T G C A T G T C A C G A T A C T G A C G T G C A T G A T C | PGR(NR)/EndoStromal-PGR-ChIP-Seq(GSE69539)/Homer | 1e-2 | -5.437e+00 | 0.0319 | 54.0 | 5.40% | 1777.3 | 3.70% | motif file (matrix) | svg |
| 61 | A T G C A G T C G C T A C G A T C G T A G C A T G C T A C G A T C T A G C A T G T G A C G T C A | CArG(MADS)/PUER-Srf-ChIP-Seq(Sullivan\_et\_al.)/Homer | 1e-2 | -5.298e+00 | 0.0361 | 58.0 | 5.80% | 1951.6 | 4.06% | motif file (matrix) | svg |
| 62 | C G A T T G C A T G C A G A T C C G T A A C T G T G A C G A C T C A T G A C T G | Tcf21(bHLH)/ArterySmoothMuscle-Tcf21-ChIP-Seq(GSE61369)/Homer | 1e-2 | -5.226e+00 | 0.0381 | 144.0 | 14.40% | 5617.4 | 11.69% | motif file (matrix) | svg |
| 63 | T G C A C T G A A T G C G T C A A C G T A T G C A C G T A C T G A C T G T G C A | ZBTB18(Zf)/HEK293-ZBTB18.GFP-ChIP-Seq(GSE58341)/Homer | 1e-2 | -5.213e+00 | 0.0381 | 89.0 | 8.90% | 3245.2 | 6.75% | motif file (matrix) | svg |
| 64 | C A T G A C G T A G T C G A T C G A T C G A T C G C T A C T A G C T A G C T A G T C A G T C G A | EBF1(EBF)/Near-E2A-ChIP-Seq(GSE21512)/Homer | 1e-2 | -5.168e+00 | 0.0392 | 116.0 | 11.60% | 4406.0 | 9.17% | motif file (matrix) | svg |
| 65 | T C G A T G A C G T A C C G T A C A G T T G A C A C G T A C T G A G C T A G C T | NeuroG2(bHLH)/Fibroblast-NeuroG2-ChIP-Seq(GSE75910)/Homer | 1e-2 | -5.124e+00 | 0.0403 | 254.0 | 25.40% | 10574.3 | 22.01% | motif file (matrix) | svg |
| 66 | C G T A A C G T A C G T A C G T A C G T A G T C A G T C C T G A A G C T A G C T | NFAT(RHD)/Jurkat-NFATC1-ChIP-Seq(Jolma\_et\_al.)/Homer | 1e-2 | -5.062e+00 | 0.0422 | 181.0 | 18.10% | 7286.9 | 15.17% | motif file (matrix) | svg |
| 67 | A T G C C T G A G A C T A C G T A C G T G T A C G A T C C G A T C T A G C A T G C G T A C G T A C T G A G A C T | STAT1(Stat)/HelaS3-STAT1-ChIP-Seq(GSE12782)/Homer | 1e-2 | -4.879e+00 | 0.0499 | 65.0 | 6.50% | 2281.2 | 4.75% | motif file (matrix) | svg |
| 68 | G C T A A G T C T A C G T G C A A T C G T C A G G C T A T C G A T C A G A G C T | ELF5(ETS)/T47D-ELF5-ChIP-Seq(GSE30407)/Homer | 1e-2 | -4.818e+00 | 0.0523 | 132.0 | 13.20% | 5158.9 | 10.74% | motif file (matrix) | svg |
| 69 | G A C T C A G T G A T C G A T C A C G T G A T C C T G A T A C G C G T A G T C A | STAT6(Stat)/Macrophage-Stat6-ChIP-Seq(GSE38377)/Homer | 1e-2 | -4.790e+00 | 0.0530 | 110.0 | 11.00% | 4203.2 | 8.75% | motif file (matrix) | svg |
| 70 | C T G A A G T C C G A T A G C T A T G C G T A C A C G T A T C G C A G T G C A T | Elf4(ETS)/BMDM-Elf4-ChIP-Seq(GSE88699)/Homer | 1e-2 | -4.662e+00 | 0.0594 | 165.0 | 16.50% | 6647.9 | 13.84% | motif file (matrix) | svg |
